# Results of Surgical Treatment of Schwannomas Arising from Extremities

**DOI:** 10.1155/2015/547926

**Published:** 2015-02-22

**Authors:** Jerzy Gosk, Olga Gutkowska, Maciej Urban, Witold Wnukiewicz, Paweł Reichert, Piotr Ziółkowski

**Affiliations:** ^1^Department of Traumatology, Clinic of Traumatology and Hand Surgery, Wroclaw Medical University, Ulica Borowska 213, 50-556 Wrocław, Poland; ^2^Division of Pathomorphology, Department of Pathomorphology, Wroclaw Medical University, Ulica K. Marcinkowskiego 1, 50-368 Wrocław, Poland

## Abstract

Schwannomas are benign neoplasms derived from Schwann cells. In this work, we present our experience in operative management of schwannomas and analyse results of treatment. Clinical material consisted of 34 patients, in whom 44 schwannomas located in extremities were excised between 1985 and 2013. Thirty-five tumours originated from major peripheral nerves and 9 from small nerve branches. Postoperatively, in the first group of tumours, pain resolved in 100%, paresthesias in 83.3%, and Hoffmann-Tinel sign in 91.6% of the patients. Improvement in motor function was noted in 28.5% of the cases, in sensory function: complete in 70%, and partial in 15%. The most frequently affected major peripheral nerves were the ulnar (11 tumours) and median (5 tumours) nerves. Schwannomas originating from small nerve branches were removed without identification of the site of origin. After their resection, definitive healing was achieved. *Conclusions*. (1) Schwannomas located in extremities arise predominantly from major peripheral nerves, most commonly the ulnar and median nerves. (2) Gradual tumour growth causes exacerbation of compression neuropathy, creating an indication for surgery. (3) In most cases, improvement in peripheral nerve function after excision of schwannoma is achieved. (4) The risk of new permanent postoperative neurological deficits is low.

## 1. Introduction

Schwannomas (neurilemmomas) are benign peripheral nerve tumours [1, 2]. They were first described by Verocay in 1908 [3]. Schwannomas usually occur in the third to fifth decades of life, with no racial and gender difference [1, 4]. They predominantly develop as solitary tumours ranging from 1.5 to 3 cm in diameter [5–7]. Presence of large tumours as well as rare cases of multiple neoplasms has been described [8–14]. Schwannomas located in the upper extremity account for 12 to 19% and in the lower extremity for 13.5 to 17.5% of all cases [15]. In the upper extremity, schwannomas are mostly located on its volar surface [1, 2, 5, 6]. Schwannomas constitute 8% of all soft tissue tumours [15, 16]. They are composed almost entirely of Schwann cells [1, 17]. The tumours are well encapsulated and characterized by their slow, noninfiltrating growth pattern [16, 18]. Slowly expanding tumour mass displaces nerve fascicles [16, 18]. Clinical symptoms that develop over time are mainly connected with compression of nerve fascicles [1, 18].

Magnetic resonance imaging (MRI) is a preferred imaging technique in the diagnosis of tumours of peripheral nervous system. On imaging scans, benign tumour of peripheral nerve presents as well-defined mass, usually fusiform in shape located within a nerve, isointense to surrounding muscles on T1-weighted images, and hyperintense on T2-weighted images [19–21]. Neurogenic tumours usually show signal enhancement after intravenous administration of contrast medium [19]. Many authors agree that it is difficult to definitively differentiate between malignant and benign neoplasms as well as between different types of benign tumours solely on the basis of MRI findings [19–23].

With regard to histopathology, schwannomas contain varying proportion of two different areas [17, 24]. Antoni type A areas are highly cellular and are composed of closely packed spindle cells which form a palisade and produce Verocay bodies. Antoni type B areas are composed of loosely arranged Schwann cells in a mucinous-like matrix [24]. One area is usually predominant over the other in every tumour [5]. There are, apart from the classical type, rarer variants of schwannoma: cellular, ancient, epithelioid, melanotic, and plexiform [3, 25–31]. Immunohistochemical analysis is useful in differential diagnosis of peripheral nerve tumours. It is performed with the use of monoclonal antibodies against proteins: S-100, CD 31, CD 34, and GFAP [17].

Treatment of choice for schwannomas is microsurgical resection [5, 6, 10–12, 18]. The aim of this work is to present our experience in operative management of schwannomas located in extremities. Our priority was to assess the obtained results of treatment and the risk of development of new, postoperative neurological deficits. Moreover, in our study, we wished to determine the most common locations of schwannomas and their sites of origin.

## 2. Material and Methods

The clinical material consisted of 34 patients of both sexes (20 females aged 22 to 81 years and 14 males aged 18 to 75 years). The patients were operated on for schwannomas located in extremities between 1985 and 2013. Mean age of the patients at the time of operation was 42.6 years.

The patients were scheduled for operation on the basis of tumour presence on clinical examination or its visualization by diagnostic imaging and concomitance of such symptoms as pain, positive Hoffmann-Tinel sign, sensory impairment, and motor deficits. Preoperative evaluation consisted of anamnesis, physical examination of the tumour, superficial sensory function testing (touch, pain, and static and dynamic sensory discrimination), muscle tone, and strength examination as well as testing for Hoffmann-Tinel sign. BMRC scale modified by Omer and Dellon was used to evaluate sensory function of the upper extremity [32, 33]. Modified and simplified Highet's classification was used for the lower extremity [34]. Muscle strength was evaluated with the use of BMRC scale [34].

The evaluation of treatment results was performed with the use of the scales mentioned above. The shortest period of postoperative follow-up has been 2 years (with the exception of 3 female patients—positions 24, 25, and 26 in Table 2, in whom it is currently 13, 14, and 12 months).

The study was approved by Local Bioethics Committee of Wroclaw Medical University.

## 3. Results

### 3.1. Location of Schwannomas

Schwannomas were located in the upper limb in 30 patients and in the lower limb in 4 patients. The tumours were located in the right side of the body in 15 patients and in the left side of the body in 19 patients. In total, 44 tumours in 34 patients were excised. Schwannomas originated from major peripheral nerves and digital nerves (35 tumours) or small nerve branches (9 tumours). Thirty-one tumours arising from major peripheral nerves were located in the upper extremity and 4 tumours in the lower extremity. Nine tumours originating from small nerve branches were located only in the upper extremity. Multiple tumours were removed in 4 patients. Two plexiform neurilemmomas arising from small nerve branches were excised from the cubital area in a 56-year-old male patient. Three tumours of the ulnar nerve at the level of the carpus were removed in a 32-year-old female patient. Six tumours were excised from digital nerves of the middle finger in a 26-year-old female and in another 22-year-old female patient 3 tumours originating from superficial radial nerve in the forearm were removed.

**Figure 1:**
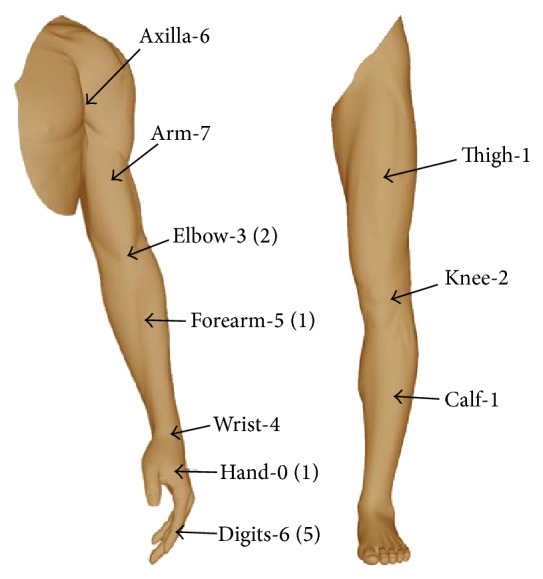
Detailed topographic distribution of schwannomas in extremities. ()—the number of tumours arising from small nerve branches is given in parentheses.

Detailed topographic distribution of schwannomas is presented in Figure 1.

Location of schwannomas arising from major peripheral nerves and sizes of resected tumours are presented in Table 1.

### 3.2. Preoperative Examination Results

In the group of tumours arising from major peripheral nerves, preoperative evaluation revealed positive Hoffmann-Tinel sign and presence of paresthesias in 24 out of 26 patients (92.3%). Pain was present in 20 out of 26 patients (76.9%). In 19 patients, pain was triggered by applying pressure to the tumour mass and appeared after exertion and one patient suffered also from night pain. Superficial sensory function impairment was detected in 20 out of 26 patients (76.9%) and motor deficit in 7 out of 26 patients (26.9%).

In the group of tumours originating from small nerve branches, presence of tumour mass with moderate pain caused by applying pressure to it and after exertion was detected during preoperative examination. Neither paresthesias nor sensory or motor deficits nor positive Hoffmann-Tinel sign was observed.

### 3.3. Histopathological Examination Results

In all of the examined tumours, histopathological pattern typical of schwannoma was observed. In the group of tumours arising from major peripheral nerves, 34 classical schwannomas (Antoni A—27, Antoni B—5, and Antoni AB—2) and one case of ancient schwannoma were diagnosed. Tumours originating from small nerve branches had histopathological pattern of classical schwannoma (Antoni A) in 6 cases, plexiform schwannoma in 2 cases, and cellular schwannoma in 1 case.

### 3.4. Postoperative Examination Results

In none of the patients tumour recurrence occurred. In the group of tumours arising from major peripheral nerves, pain resolved in 20 patients (100%). No relationship between presence of pain and the size of tumour has been observed. Paresthesias resolved in 20 out of 24 patients who had suffered from them preoperatively (83.3%). Negative Hoffmann-Tinel sign was observed in 22 out of 24 patients, in whom it had been positive before operative treatment (91.6%). Sensory deficits completely resolved in 14 out of 20 patients (70%) and partial improvement in sensory function was noted in 3 out of the remaining 6 patients who had reported sensory impairment preoperatively (15%). Improvement in motor function was observed in 2 out of 7 patients (28.5%).

Detailed summary of clinical material comprising tumours originating from major peripheral nerves is presented in Table 2.

In postoperative course, new neurological deficits were observed in 3 patients (Table 2—numbers 16, 22, and 26). New sensory deficit was observed in a 38-year-old male after removal of schwannoma (tumour measuring 3.5 cm in diameter excised without damage to fascicular structure) from the ulnar nerve at the level of axilla. In a 35-year-old female, new motor deficit was observed after excision of schwannoma (tumour measuring 4.0 × 3.0 × 3.0 cm removed with transection of 2 fascicles) from radial nerve at the level of axilla. In a 51-year-old female, significant increase in intensity of paresthesias after excision of schwannoma (tumour measuring 3.5 × 3.0 × 1.5 cm removed without damage to fascicular structure) from the medial cutaneous nerve of the arm was observed.

The tumours arising from small nerve branches were removed in one piece without identification of the site of origin. After removal of those tumours, definitive healing was achieved.

## 4. Discussion

In our patients, schwannomas were predominantly located in the upper extremity (40 out of 44 excised tumours). Schwannomas originated mainly from major peripheral nerves (35 tumours) and the most common sites of their origin were the following nerves: ulnar, median, and radial nerves. This is in agreement with results obtained by other authors [5, 35–39]. Having analysed data from 3 clinical centres comprising 72 schwannomas, Siqueira et al. described in the upper extremity 12 cases of tumour location in ulnar nerve, 12 cases of tumour location in median nerve, and 3 cases of tumour location in radial nerve [37]. In clinical material consisting of 36 schwannomas, Date et al. observed tumours of the upper extremity in descending order of frequency in ulnar, median, and radial nerves [38]. These authors described also 2 cases of rare location of schwannoma in musculocutaneous nerve [38]. Having analysed clinical material comprising 24 cases of schwannoma located in the upper extremity, Adani et al. established, as the most common sites of tumour origin, the following nerves (in descending order of frequency): ulnar nerve (14 tumours), median nerve (4 tumours), musculocutaneous nerve (3 tumours), and digital nerves (3 tumours) [5]. In our patients, none of the tumours were located in musculocutaneous or axillary nerve. The latter location is a subject of casuistic reports [40]. Out of rare schwannoma locations in our study, we wish to highlight 2 cases of tumours located in medial cutaneous nerve of the arm.

Tumours arising from small nerve branches made up 20.4% of the total number of removed schwannomas (9 out of 44 tumours). All of them were located in distal portions of the limbs, whereas schwannomas originating from major peripheral nerves were evenly distributed along the length of the upper limb. Schwannomas mostly develop as solitary tumours [5–7]. Analysis of our material is consistent with the above result reported previously by other authors. In 30 patients, presence of a solitary schwannoma was detected (88.2%). In the remaining 4 patients, a total of 14 tumours were removed. None of these subjects manifested symptoms of neurofibromatosis type 2. Examinations performed in each of the cases enabled diagnosis of segmental sporadic schwannomatosis. Two of these cases have been described in a separate article.

Conclusive diagnosis of the structure of the excised tumours was made on the basis of histopathological examination. Classical highly cellular pattern (Antoni A) was dominant in both groups. Out of rare schwannoma types, one case of ancient schwannoma located in superficial radial nerve was diagnosed among our patients. A separate article has been devoted to this particular case. In the group of tumours originating from small nerve branches, 2 tumours showed growth pattern of plexiform schwannoma and 1 of cellular schwannoma. Plexiform schwannoma is a rare variant accounting for about 5% of all schwannomas [41]. It can be associated with neurofibromatosis type 2 or schwannomatosis; however, unlike plexiform neurofibroma, it is not linked to neurofibromatosis type 1. Similarity between these 2 types of tumours is based on their multinodular growth pattern [41]. Cellular schwannoma shows features of nuclear atypia. Because of its mitotic activity, cellular schwannoma may be diagnosed as malignant tumour [27].

Because of their eccentric, noninfiltrating growth, schwannomas can often be excised without or with only slight damage to fascicular structure [5, 16, 18, 42]. In the article published by Adani et al. in 2008, comprising 24 schwannomas located in the upper extremity, in 20 cases the authors were able to remove tumours without damage to the fascicular structure [5]. Likewise, in our series of cases out of the total number of 35 tumours located in major peripheral nerves, 16 were excised without damage to fascicular structure and 11 with transection of one thin fascicle.

Possibility of atraumatic tumour removal does not eliminate the risk of developing new postoperative neurological deficits. Their causative factors can be diverse [43–45]. Firstly, the possibility of damage to fascicles encircling the tumour during incision of epineurium must be considered [44]. Nerve function impairment may also result from irritation or compression of healthy fascicles during dissection of the tumour. According to Park et al., transection of fascicles entering the tumour may result in postoperative neurological deficits [43]. Donner et al. expressed a contrary opinion on this topic. The above authors have determined, based on intraoperative stimulation, that fascicles entering the tumour are usually nonfunctional and their transection does not cause additional neurological deficits [45]. Incidence of neurological deficits after removal of schwannoma varies between 1.5 and 80 percent [5, 10, 12, 37, 43–45]. Significantly high percentage of complications occurs in short-term observations. Adani et al. in early postoperative period described worsening of paresthesias in 23 out of 24 patients [5]. They spontaneously resolved in all of the patients within 12 months from the operation [5]. Siqueira et al. reported postoperative complication rate of 15.2% (11 out of 72 operated-on patients) [37]. The majority of the reported deficits were transient. Eventually, permanent sensory impairment occurred in 3 patients and permanent motor deficit in 1 patient [37]. In clinical material presented by Kim et al. comprising 30 schwannomas located in the lower extremity, complication rate in the early postoperative period was 76.7%. Ultimately, major neurological deficit persisted only in 2 of the operated-on patients [12]. Similarly, in the article published by Kang et al. in 2000, permanent sensory impairment was present in 1 out of 20 patients [10]. Knight et al. observed serious postoperative complications in 5 out of 198 treated patients [46]. In our cases in early postoperative period, new neurological deficits were observed in 3 out of 26 patients with tumours located in major peripheral nerves (11.5%). These symptoms resolved in all of the patients within 6 months' follow-up.

During final clinical evaluation of our patients, we observed no new permanent neurological deficit; at most there was no improvement in certain neurological functions that had been impaired during preoperative assessment. The least evident improvement was seen in motor function. Motor deficit was observed in 7 out of 26 patients (26.9%) at baseline. It has been confirmed by several studies that schwannomas can rarely induce impaired motor function [7]. Neural tumours producing motor deficit should always raise a high suspicion of malignancy [47]. In our opinion, patients with benign tumours may also show motor impairment preoperatively.

Extracapsular excision is an operative technique commonly used for removal of schwannomas [11, 12, 38, 48]. To reduce the risk of damage to nerve fascicles during dissection of a tumour, different authors suggest modifications of the operative technique. Hussain et al. propose “tumour release by incising the capsule far lateral to the path of the nerve and dissecting the tumour circumferentially from its capsule. The epineural capsule is then left behind and acts as a protective covering of the nerve” [48]. The intracapsular technique was used by Date et al. [38]. The above authors have compared results obtained after extracapsular and intracapsular enucleation of schwannoma and have found the latter technique to be superior due to lower risk of complications [38]. In case of presence of adhesions between epineurium and the capsule, Date and co-workers performed microenucleation of the tumour (“tumour was resected piece-by-piece”), unlike Hussain et al. who removed the tumour in one piece [38, 48].

**Figure 3:**
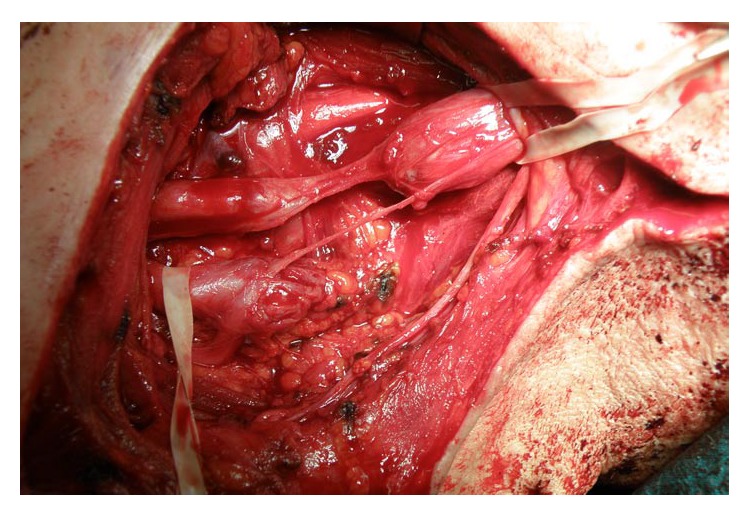
MJ, age 51, male. Previously operated on in another hospital for schwannoma of the upper trunk of the brachial plexus. Intraoperative view: iatrogenic damage to the upper and middle trunks of the brachial plexus after excessively radical excision of the tumour with a part of healthy neural tissue.

When neurological deficits after excision of schwannomas are considered, awareness must be raised that every surgical intervention in a peripheral nerve is connected with the risk of complications. However, as the analysis of our clinical material and the review of literature revealed, in most cases improvement in nerve function is achieved and onset of new persistent postoperative deficits is limited to isolated cases [5, 10, 12, 37, 46]. At the same time, it must be realized that growth of tumour causes exacerbation of symptoms of compression neuropathy [37]. It has been suggested that slow growth pattern of schwannomas allows for adaptation of nerve function to the pressure effects [7]. However, at a certain point in their growth, most tumours become symptomatic [1, 2, 5, 6, 18]. In our material, only patients in whom tumour growth caused obvious clinical symptoms were scheduled for operation. In our opinion, chances for nerve function improvement after excision of tumour justify the risk related to surgical treatment. As a result of operative treatment, the authors of this work achieved improvement in nerve function in most of the patients. Operative removal of peripheral nerve tumours requires from the surgeon both microsurgical experience and knowledge on mechanisms of tumour development. Lack of this knowledge and adequate experience in treatment of this type of lesions can result in serious iatrogenic damage to nerves (Figure 3).

## 5. Conclusions

1. Schwannomas located in extremities arise predominantly from major peripheral nerves, with the most common nerve of origin being the median nerve and the ulnar nerve.
2. The risk of exacerbation of compression neuropathy caused by gradual tumour growth justifies surgical intervention in symptomatic peripheral nerve tumours.
3. With the use of adequate operative techniques, in most cases, improvement in peripheral nerve function after the excision of schwannoma is achieved.
4. The risk of new permanent postoperative neurological deficits is low.
5. Schwannomas originating from small nerve branches are usually diagnosed postoperatively on the basis of histopathological examination results.

## Figures and Tables

**Figure 2:**
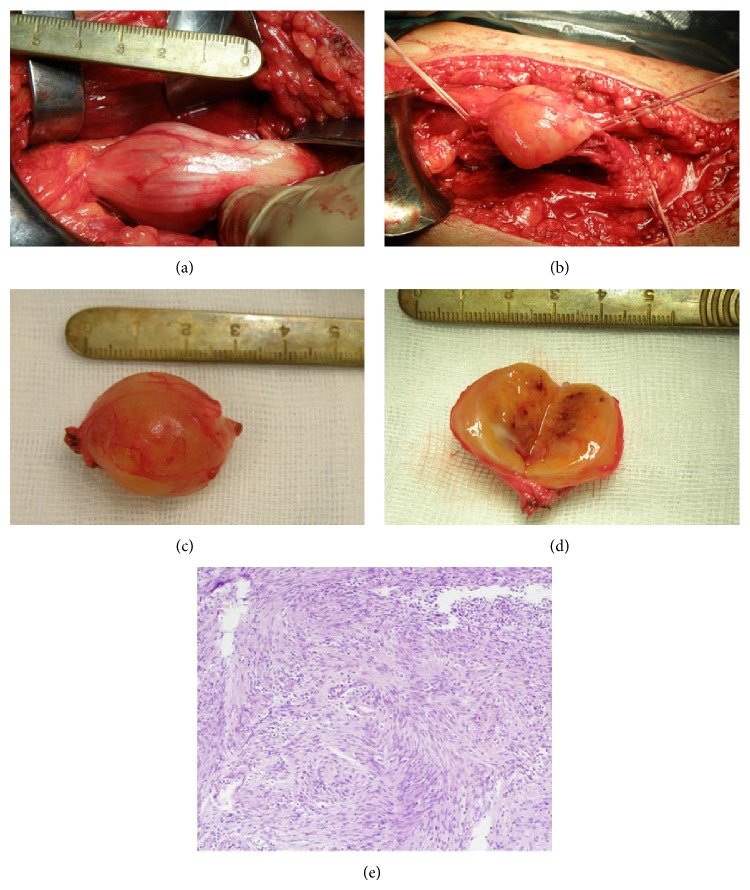
HJ, age 46, male. Operated on 16.12.2010—removal of a tumour of left sciatic nerve in the thigh. (a) Intraoperative view: exposure of the tumour; (b) intraoperative view: status after dissection of the tumour; (c) postoperative view: appearance of the tumour after resection; (d) postoperative view: cross-section of the tumour; (e) histopathological image of the tumour. H&E stain. Magnification ×100. Examination result (number 422104 from 27.12.2010)—schwannoma Antoni A.

**Table 1:** Location of schwannomas arising from major peripheral nerves and sizes of resected tumours.

Number	Location	Number of patients	Number of tumours	Size of tumours
1	Ulnar nerve	9	11	From 1.0 × 0.5 × 0.5 cm to 18.0 × 1.5 × 1.0 cm
2	Median nerve	5	5	
3	Radial nerve	3	3	
4	Medial cutaneous nerve of the arm	2	2	
5	Superficial radial nerve	1	3	
6	Deep radial nerve	1	1	
7	Digital nerves	1	6	
8	Superficial peroneal nerve	2	2	
9	Common peroneal nerve	1	1	
10	Sciatic nerve^*^	1	1	
11	Small nerve branches	9	9	From diameter of 0.5 cm to 6.0 × 3.0 × 1.0 cm

^*^See Figure 2.

**Table 2:** Detailed summary of clinical material comprising tumours originating from major peripheral nerves.

Case number	Location	Nerve^1^	Type of operation^2^	Pain^3^		Hoffmann-Tinel sign		Paresthesias		Sensory deficit^4^		Motor deficit	
				Pre^*^	Post^**^	Pre^*^	Post^**^	Pre^*^	Post^**^	Pre^*^	Post^**^	Pre^*^	Post^**^
1	Arm	Median	T-1	0	0	+	−	+	−	+/S_2+_	+/S_3_	−	−
2	Arm	mca	T-1	0	0	+	−	+	−	+/S_2+_	−	−	−
3	Knee	cp	RG (4 × 18 cm)	C	0	+	+	+	+	+/S_0_	+/S_1_	+/M_0_	+/M_0_
4	Calf	sp	T-1	C	0	+	−	+	−	+/S_2_	−	+/M_3_	−
5	Axilla	Radial	T-1	0	0	−	−	−	−	−	−	+/M_3_	−
6	Wrist	Ulnar (3t)	ND	0	0	+	−	+	−	+/S_2+_	−	−	−
7	Arm	Ulnar	T > 2	C	0	+	−	+	−	+/S_0_	+/S_0_	+/M_0_	+/M_0_
8	Axilla	Ulnar	ND	C	0	+	−	+	−	+/S_2+_	−	−	−
9	Arm	Ulnar	ND	0	0	+	−	+	−	+/S_2+_	−	−	−
10	Arm	Median	ND	C	0	+	−	+	−	+/S_2+_	−	−	−
11	Forearm	Median	T > 2	C, N	0	+	−	+	−	+/S_2+_	+/S_3_	+/M_3_	+/M_3_
12	Elbow	Ulnar	ND	C	0	+	−	+	−	+/S_2+_	−	−	−
13	Axilla	Ulnar	T-1	C	0	+	−	+	−	+/S_3+_	−	−	−
14	Arm	Radial	T-1	C	0	+	−	+	−	+/S_3+_	−	−	−
15	Forearm	Median	T-2	C	0	+	−	+	−	+/S_3+_	−	−	−
16	Axilla	Ulnar	ND	C	0	+	−	+	+^!^	−	+/S_3+_^!^	−	−
17	Wrist	Median	ND	C	0	+	−	+	−	+/S_3+_	−	−	−
18	Arm	Ulnar	T-1	C	0	+	−	+	−	+/S_3+_	−	−	−
19	Elbow	Ulnar	RG (2 × 5 cm)	C	0	+	+	+	+	+/S_3_	+/S_3_	+/M_3_	+/M_3_
20	Thigh	Sciatic	T-2	C	0	+	−	+	−	+/S_2_	−	−	−
21	Knee	sp	T-1	C	0	+	−	+	−	+/S_2_	−	−	−
22	Axilla	Radial	T-2	C	0	+	−	+	−	+/S_3_	+/S_3_	−	+/M_4_^!^
23	Elbow	dr	DN	0	0	−	−	−	−	−	−	+/M_3_	+/M_3_
24	Forearm	sr (3t)	T-1/ND	C	0	+	−	+	−	−	−	−	−
25	Hand	Digital (6t)	ND	C	0	+	−	+	−	−	−	−	−
26	Axilla	mca	T-1	C	0	+	−	+	+^!^	−	−	−	−

^1^Nerve: mca: medial cutaneous nerve of the arm, cp: common peroneal nerve, sp: superficial peroneal nerve, dr: deep radial nerve, and sr: superficial radial nerve.

^2^Type of operation: ND: tumour excision without damage to fascicular structure, T-1: tumour excision with transection of 1 fascicle, T-2: tumour excision with transection of 2 fascicles, T > 2: tumour excision with transection of more than 2 fascicles, RG: tumour excision and reconstruction with grafts, and DN: tumour excision and reconstruction with direct neurorrhaphy.

^3^Pain: C: pain during compression and after exertion, N: night pain, and 0: no pain.

^4^Sensory deficit: upper extremity: assessed according to BMRC scale modified by Omer and Dellon and lower extremity: assessed according to Highet's scale.

^!^New postoperative deficits.

^*^Preoperative.

^**^Postoperative.
